# Visual, spectral, and microchemical quantification of crystalline anomalies in otoliths of wild and cultured delta smelt

**DOI:** 10.1038/s41598-022-22813-w

**Published:** 2022-12-01

**Authors:** Levi S. Lewis, Jonathan L. Huang, Malte Willmes, Rachel A. Fichman, Tien-Chieh Hung, Luke T. Ellison, Troy A. Stevenson, Swee J. Teh, Bruce G. Hammock, Andrew A. Schultz, John L. Grimsich, Magdalena H. Huyskens, Qing-Zhu Yin, Leticia M. Cavole, Nicholas W. Botto, James A. Hobbs

**Affiliations:** 1 University of California, Davis, USA; 2 University of California, Santa Cruz, USA; 3 United States Fish and Wildlife Service, Vernal, UT, USA; 4 University of California, Berkeley, USA

**Keywords:** Limnology, Geochemistry, Ichthyology

## Abstract

Developmental abnormalities in otoliths can impact growth and survival in teleost fishes. Here, we quantified the frequency and severity of developmental anomalies in otoliths of delta smelt (*Hypomesus transpacificus*), a critically endangered estuarine fish that is endemic to the San Francisco Estuary. Left–right asymmetry and anomalous crystalline polymorphs (i.e., vaterite) were quantified and compared between wild and cultured populations using digital image analysis. Visual estimates of vaterite were validated using X-ray diffraction, Raman spectroscopy, laser ablation ICPMS, and electron probe microanalysis. Results indicated that cultured delta smelt were 80 times more likely to contain a vateritic otolith and 18 times more likely to contain relatively large (≥ 15%) amounts of vaterite. Similarly, cultured fish exhibited 30% greater asymmetry than wild fish. These results indicate that cultured delta smelt exhibit a significantly higher frequency of vestibular abnormalities which are known to reduce fitness and survival. Such hatchery effects on otolith development could have important implications for captive culture practices and the supplementation of wild fish populations with cultured individuals.

## Introduction

Otoliths, or “ear stones”, are calcified structures in teleost fishes that perform critical sensory functions including hearing, balance, and acceleration^1^. As biominerals, otoliths are composed primarily of calcium carbonate (CaCO_3_) embedded with organic molecules (e.g., polysaccharides and proteins), are metabolically inert, and grow continuously through the daily accretion of concentric layers of otolith matrix^2,3^. The biomineralization of CaCO_3_ also incorporates a variety of elements from the ambient water, thus providing a record of a fish’s environmental and metabolic history^4–6^. Therefore, otoliths serve as permanent records of the age, growth rate, and life history of fishes, thus providing useful data for informing the management of fisheries and ecosystems^2,7^.

Three pairs of otoliths (sagittae, lapillae, and asterisci) are present within a fish’s cranium, with each pair being comprised of varying amounts of three different CaCO_3_ polymorphs: aragonite, vaterite, and calcite^8^. Within teleost fishes, sagittae and lapilli are often comprised primarily of aragonite whereas asterisci are composed primarily of vaterite, and calcite is occasionally present in smaller amounts^2,9^. Unlike teleosts, sagittal otoliths of basal groups of fishes (e.g., sturgeons and paddlefishes of the order Acipenseriformes) are composed primarily of vaterite^10^. Despite these general patterns, the makeup of otoliths can vary considerably within taxonomic groups and even among individuals within a species^8^. In teleosts, aragonitic sagittae and lapilli are often the largest otoliths and are, therefore, most commonly used in age, growth, and geochemical analyses.

Several developmental abnormalities in otoliths have been described including asymmetry^11,12^ and crystalline anomalies^13–15^. In particular, the anomalous replacement of aragonite by vaterite in sagittae and lapillae of many teleosts causes these “vateritic” otoliths to become less dense, less stable, and more transparent than usual^16,17^. Such vateritic otoliths have been observed in many species such as lake trout (*Salvelinus namaycush*)^18^, Atlantic herring (*Clupea harengus*)^16^, Atlantic salmon (*Salmo salar*)^19^, and Chinook salmon (*Oncorhynchus tshawytscha*)^13,20^. Vateritic otoliths are most common in hatchery-reared populations of fishes, with up to 50% of otoliths in hatchery fish containing vaterite (versus 8% in wild fish), and with vaterite compositions of up to 91%^16,21^.

The effects of vestibular abnormalities in fishes remain poorly understood. However, anomalies in otoliths have been associated with acute and chronic stress^22,23^ and are known to result in reduced function, thus potentially negatively impacting fitness by impairing foraging, navigation, and predator avoidance^21,24–28^. Moreover, vateritic otoliths often exhibit a loss of otolith microstructures and alteration of relative elemental concentrations, including enrichment in Mn/Ca and Mg/Ca, and depletion in Sr/Ca, Ba/Ca, and Na/Ca; thus complicating age, growth, and life-history reconstructions based on otolith analyses^8,18,29–31^. Therefore, studies quantifying otolith compositions and sources of variation in minerology are needed in order verify interpretations of otolith-based metrics^32^.

The delta smelt (*Hypomesus transpacificus*) is an estuarine forage fish that is endemic to the San Francisco Estuary (SFE). This migratory zooplanktivore is adapted to cool, turbid, low-salinity habitats^33,34^ and exhibits a complex life history that allows it to exist within the dynamic low-salinity habitats of the SFE^35^. Although historically abundant, its population has declined to < 1% of 1980s levels, leading to its listing as threatened by the federal Endangered Species Act (ESA)^36^, endangered by the state ESA^37^, and critically endangered by the International Union for Conservation of Nature^38^. This population decline is likely due to multiple human impacts including hydrological modifications resulting from dams and exports, direct entrainment in water diversions, pollution, invasive species, trophic collapse, and climate change^34^. Municipal and agricultural demands for freshwater have made conservation of this imperiled fish a difficult and controversial issue in California politics and resource management^39^.

To conserve delta smelt, a captive culture program has been established at the Fish Conservation and Culture Laboratory, UC Davis, that produces 20,000–50,000 delta smelt each year to support experiments, serve as a reserve population, and provide cultured fish that can be released to supplement the remaining wild population^40,41^. However, the effects of hatchery conditions and domestication on the fitness of cultured delta smelt remain poorly understood^42^. For example, little is known about the comparative health and survival of cultured versus wild individuals, and such differences could influence the outcomes of experiments and supplementation efforts using cultured fish. Similarly, otolith-based analyses have been used to describe environmental effects on growth^33^ and life history^35,43,44^ of wild delta smelt; however, the prevalence and potential effects of crystalline anomalies have not been examined. Studies that quantify developmental abnormalities in otoliths, therefore, are important for examining differences in health and fitness among wild and cultured fish populations and for validating the interpretation of otolith-based metrics.

Several approaches can be used to identify variation in the crystalline structure of CaCO_3_ in otoliths. Visual estimates based on digital image analysis are the most efficient approach; however, such approaches should first be validated using other techniques. Analysis of bulk (mass %) of CaCO_3_ polymorphs in otoliths can be estimated using X-ray powder diffraction (XRD), whereas spatial distributions in otoliths can be identified using Raman spectroscopy or laser-ablation inductively coupled plasma mass spectroscopy (LA-ICPMS), each being applied across polished otolith surfaces. XRD is a destructive technique that examines the X-ray scattering pattern of a crushed (powdered) crystalline sample to identify the relative abundances of different polymorphs^14,45^. Raman spectroscopy is a non-destructive technique using excitation by a laser light source and unique spectral shifts to identify spatial patterns in the molecular structure of substrates^18,46^. LA-ICPMS is a minimally destructive technique that uses a focused laser to ablate a thin surface layer of material from the otolith, with the ablated particles passed to a coupled plasma mass spectrometer to determine their chemical composition. Thus, LA-ICPMS can be used to quantify spatial patterns in the elemental composition of otolith sections, with known chemical variation among different crystalline polymorphs^29^.

Here, we used an interdisciplinary approach to quantify and contrast vestibular abnormalities, including asymmetry and the presence of irregular CaCO_3_ crystalline structure (e.g., vaterite) in sagittal otoliths of delta smelt. Specifically, we validated digital image analysis using a combination of XRD, Raman Spectroscopy, and LA-ICPMS geochemistry, and then applied this tool to contrast otolith developmental anomalies between wild and cultured delta smelt populations. We also explored the potential effects of hatchery rearing conditions (e.g., food availability and temperature) on otoliths of the cultured population. By quantifying developmental abnormalities in otoliths of cultured and wild delta smelt populations, we aimed to advance the interpretation of otolith-based metrics and to improve our understanding of the relative health and survival of the cultured population, with important implications for hatchery production and supplementation of the wild population of this critically endangered species.

## Methods

### Sample collection

This study took advantage of archived delta smelt otoliths from previous work conducted as part of the delta smelt Captive Culture Program at the UC Davis Fish Conservation and Culture Laboratory (FCCL). Prior work was carried out in accordance with relevant guidelines and regulations under the UC Davis Institutional Animal Care and Use Committee Protocols #18071 and #19841, with methods reported in accordance with ARRIVE guidelines. In short, cultured (F11) delta smelt were hatched and reared at the FCCL following established protocols^41,47^. Fish were spawned and reared in freshwater (salinity of 0.4 ppt) at 16 °C for 120 days (~ 50 mm total length), then individually tagged using Visible Implant Alphanumeric (VIA) tags, and randomly distributed among four tanks (88 specimens/tank) with varying temperatures (14 °C or 18 °C) and varying feed (ad libitum or no feed). This resulted in four distinct treatments: 14 °C fed, 14 °C unfed, 18 °C fed, 18 °C unfed. Fish were subsequently monitored for 70 days. After the treatment period, fish were euthanized following approved veterinary procedures using 500 mg/L buffered tricaine methanesulfonate (MS-222) and were then archived in a freezer at − 20 °C.

For the present study, otoliths were removed from archived specimens and stored in 95% ethanol. Upon dissection, significant vaterite could be observed in several otolith samples, thus providing an opportunity to quantify the prevalence of vaterite in cultured delta smelt. Results for cultured fish were compared to those of 104 wild delta smelt that were previously collected in 2019 by the US Fish and Wildlife Service’s Enhanced delta smelt Monitoring (EDSM) Kodiak trawl survey^48^ (Table 1). Otoliths of wild delta smelt were previously dissected and archived following the same protocols as used for cultured fish. Sex was not included in the analysis because all fish were less than 1 year of age, and delta smelt do not exhibit sexual dimorphism^49^. The transfer and processing of wild and cultured delta smelt otoliths was approved by the California Department of Fish and Wildlife (CDFW) under a Section 2081a Memorandum of Understanding to L. Lewis (No. 2021-0004-R3_Lewis).

**Table 1:** Numbers of cultured and wild delta smelt that were assigned to each of the four vaterite categories (I–IV). Fish were assigned based on the most vateritic otolith.

Category	Vaterite (max %)	Cultured (n)	Wild (n)
I	< 1.0	38	103
II	1.0–14.9	59	1
III	15.0–29.9	13	0
IV	≥ 30.0	9	0
Total fish	0–67.6	119	104

### Otolith preparation

Whole otolith images were taken sulcus side down with an AmScope MU1000 10MP camera on a Leica SteroZoom7 dissecting scope at 20 × or 30 × magnification with a scale bar (1 mm increments). After imaging, otoliths were mounted to glass microscope slides with a thermoplastic glue (Crystalbond) and stored in plastic microscope slide boxes. A subset of otoliths (n = 24) with varying proportions of vaterite were selected to quantify whole otolith polymorph composition using XRD. Otoliths selected for XRD were cleaned with 99.5% acetone to remove any Crystalbond, and were then sonicated in MilliQ water, dried, and stored in clean glass vials prior to analysis. An additional subset of five samples, representing a broad range of vaterite compositions, were selected and prepared for analysis by Raman spectroscopy and laser ablation. These samples were sanded on the sulcus side with 600, 800, and 1200 grit sandpaper and polished using a polishing cloth wetted with 0.3-µm alumina polish. Polished sections were sonicated a second time in MilliQ water prior to analysis.

### Digital image analysis

**Figure 1:**
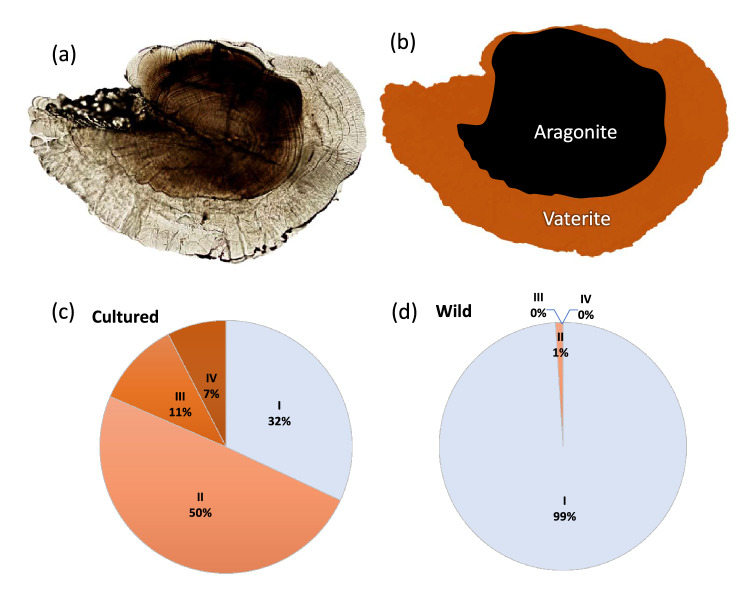
Quantifying vaterite in delta smelt otoliths. (**a**) Transmitted light image of a sagittally polished Category IV otolith exhibiting vaterite (transparent area). (**b**) Segmentation of aragonitic and vateritic sections of the otolith. The proportions of delta smelt assigned to each vaterite category (I–IV, see Table 1) are provided for the (**c**) cultured and (**d**) wild populations.

Left and right otoliths from a total of 119 cultured and 104 wild delta smelt were included in digital image analyses (Fig. 1, Table 1), with fish containing a broken or missing otolith being previously excluded (n = 16). Image analysis was used to quantify several otolith metrics using ImageJ (Version 2.0)^50,51^. Differences in the size of left and right otoliths were used to estimate otolith asymmetry for each fish following Eq. (1), where *DV*_*L*_ and *DV*_*R*_ are the maximum dorsoventral measurements (in µm) for the left and right otolith, respectively.

1 $$\text{Asymmetry } ({\%}) = \frac{|({DV}_{L}-{DV}_{R})|}{{DV}_{R}} \times 100.$$

The total surface area (*A*_*tot*_, in µm^2^) of each otolith was measured from a calibrated digital image using the ‘Analyze Particle’ operation in ImageJ^52^. Aragonitic and vateritic segments of each otolith image were then identified visually based on differences in texture and translucency, with vaterite appearing more transparent. The area of aragonite (*A*_*arag*_), typically in the center of the otolith, was then measured by drawing a polygon over the aragonitic area using the ‘Freehand’ operation in ImageJ. The surface area of vaterite (*A*_*vat*_) in each otolith was calculated by subtracting *A*_*arag*_ from *A*_*tot*_. Vaterite “prevalence” (percent abundance based on area) was then calculated according to Eq. (2). Vaterite prevalence was quantified for each otolith of each fish, and the most vateritic otolith (‘max’) used to classify each fish into 1 of 4 vaterite categories: I (< 1.0%), II (1–14.9%), III (15–29.9%), IV (> 30.0%) (Fig. 1, Table 1).

2 $${\text{Vaterite Prevalence }} ({\%})= \frac{{A}_{vat}}{{A}_{tot}} \times 100$$

### Raman spectroscopy

Raman spectroscopy was used to validate results from digital image analysis with respect to spatial patterns of vaterite and aragonite in delta smelt otoliths. Raman spectroscopy is a non-destructive technique using excitation by a laser light source and the unique spectral shifts of backscattered light, due to specific bond vibrations, to identify different molecules and their crystalline microstructures^18,46^. This approach provides discrete spatial observations across 2-dimensional (polished) surfaces. Each measurement yields a Raman spectrum, comprised of numerous peaks, with each peak reflecting a Raman Shift (in wavenumber, cm^−1^) due to the substrate’s molecular composition. Based on values provided in the literature^18,46^, we examined multiple peaks that could be used to discriminate between the three CaCO_3_ polymorphs commonly found in otoliths: aragonite (1085 and 705 cm^−1^), vaterite (267, 300, 740, 750, 1075, and 1090 cm^−1^), and calcite (282 and 711 cm^−1^).

Raman analyses were performed at the Keck Spectral Imaging Facility, UC Davis, using a Renishaw Confocal Raman Microscope equipped with a diode laser with an excitation peak of 785 nm at 0.37 mm working distance, a 50 × objective, and a grating of 1800 mm^−1^, calibrated to a peak of 520 cm^−1^ on a silicone standard. Spectra were collected with an integration time of 10 s and a peak range of 130–1200 cm^−1^. Five otoliths were selected for Raman spectroscopy, spanning the full range of vaterite categories (I–IV). Multiple spots were analyzed along a transect from the otolith core to the ventral edge: (1) aragonite, (2) aragonite edge, (3) vaterite edge, (4) vaterite, with additional radial transects conducted in the most vateritic (category IV) otolith (Fig. 3). Peaks were automatically detected in each spectrum using the “findpeaks” function in the *pracma* package^18,53^ in R (v. 3.6.2) and confirmed graphically. The peaks are identified relative to background counts, which are determined by the sample type and machine settings and therefore have arbitrary units. Additional peaks not known to be CaCO_3_ polymorphs were observed due to contamination by binding resin^54^ and were thus excluded.

### X-ray diffraction

The bulk percent composition of vaterite in otoliths was measured using X-ray diffraction (XRD). XRD is a destructive technique that examines the X-ray scattering pattern of a crushed (powdered) crystalline sample to identify the relative abundances of different polymorphs^14,45^. A total of 24 previously imaged otoliths from cultured delta smelt, spanning the range of vaterite categories (I–IV), were selected for XRD analysis. Each otolith was then crushed to a fine powder and scanned for 80 min on a PANalytical X’Pert Pro diffractometer equipped with a PW3064 sample spinner and Co X-ray tube X’Celerator detector using a scan range of 20°–70° 2*θ* with a step size of 0.0017° 2*θ*. All XRD analyses were performed at the X-Ray Diffraction Lab, UC Berkeley, CA. To validate visual estimates of vaterite, vaterite prevalence based on XRD (bulk %) was contrasted with estimates based on image analysis using linear regression.

### LA-ICPMS

Elemental concentrations in otoliths were measured using a Photon Machines 193 nm ArF Excimer laser with a HelEx dual-volume LA cell coupled to a Thermo Element XR HR-ICPMS in the Yin Lab at the Department of Earth and Planetary Sciences at UC Davis. The repetition rate of the laser was set at 10 Hz and fluence was ~ 3 J cm^−2^. A line of spots was ablated from the edge to the core with a spot size of 40 µm and a spacing of 40 µm. Before data collection, a cleaning run (pre-ablation) was performed across the same trajectory with a larger (80 µm) spot size. We measured a large suite of potentially informative analytes including lithium, sodium, magnesium, potassium, calcium, manganese, zinc, strontium, and barium (^7^Li, ^23^Na, ^24^Mg, ^39^K, ^43^Ca, ^44^Ca, ^55^Mn, ^66^Zn, ^88^Sr, and ^138^Ba, respectively). Data were reduced with the Trace Element data reduction schema in the Iolite^55^ software package, with NIST 612 as the standard reference material and ^43^Ca as the internal standard. Otoliths were assumed to contain 38.8 wt% Ca^56^. NIST 610 and 612 glasses were measured prior to and between each sample and used as external reference materials to correct for instrument drift. Element-specific limits of detection were calculated as 3 times the standard deviation of the element-specific background value. Measured concentrations of an element below its respective detection limit were set to zero, indicating negligible abundance.

### Electron microprobe

Relative abundances of elements were mapped spatially across the surface of a category IV vateritic otolith using a Cameca SX-100 electron microprobe (Earth and Planetary Sciences, UC Davis) equipped with five WDS spectrometers and a tungsten thermionic emission filament. The WDS spectrometers were tuned to Sr, Ca, Ba, and Mg X-ray lines, with backscatter electron images captured for both compositional and topographical modes. An operating voltage of 15 kV was used, with the beam current regulated at 100 nA with a fully focused beam. The beam was fixed in place and the stage was scanned to capture spatially accurate maps. The maps were acquired as mosaic cells and reconstructed into a single imagen using the Cameca PeakSight 6.5 software. The software was used to adjust the scales of the image for clarity, and color LUTs were applied as well.

### Statistical analyses

Estimates of vaterite composition based on image analysis and XRD were compared by linear regression. Linear models were used to compare vaterite composition and asymmetry among cultured and wild fish. The presence of vaterite was compared between cultured and wild fish by logistic regression. The potential effects of recent hatchery conditions on vaterite and asymmetry in cultured fish were each examined using linear mixed effect models that included temperature, feed, and their interaction as fixed effects and tank as a random effect. Logistic regression was used to assess potential effects of hatchery conditions on vaterite presence. All analyses and plotting were conducted using R statistical analysis software (v. 3.6.2)^57^.

## Results

### X-ray diffraction

**Figure 2:**
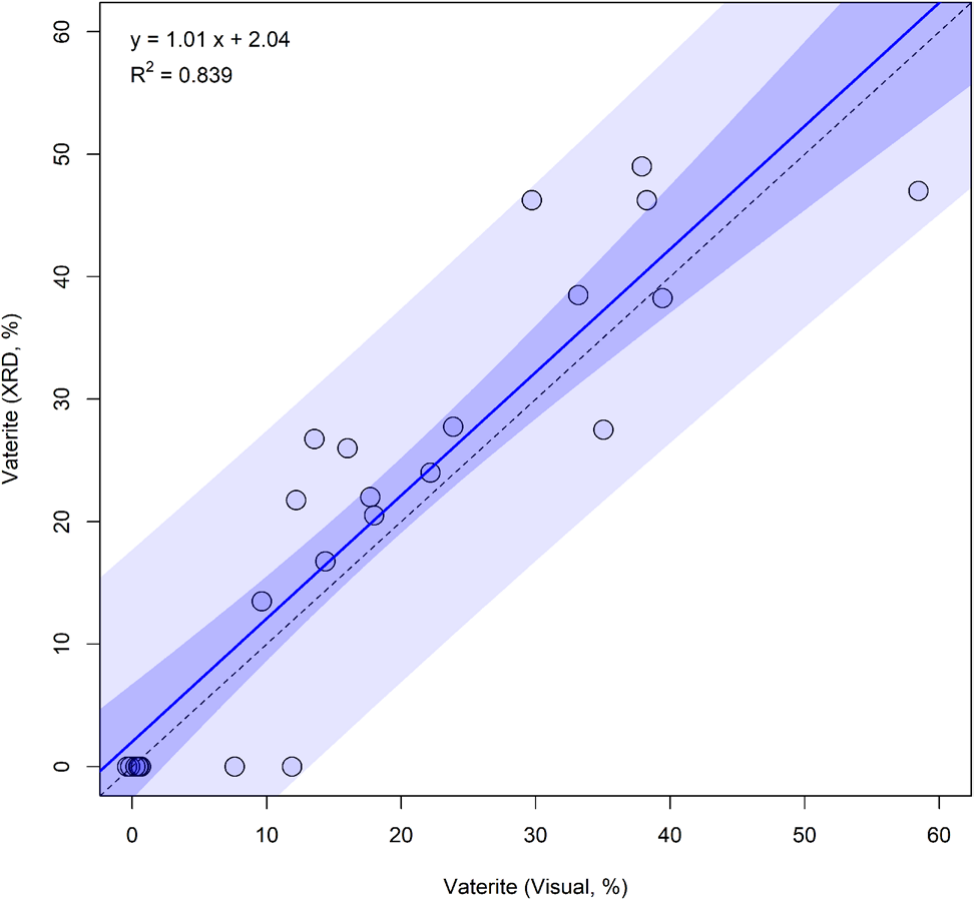
Scatter plot and results of a linear model contrasting the estimates of vaterite prevalence based on X-ray diffraction (XRD) and digital image analysis. A 1:1 line (dashed line) and fitted line (solid line) are shown with the 95% confidence interval (dark blue) and 95% prediction interval (light blue).

Comparison of vaterite prevalence based on XRD and image analysis indicated a strong linear positive relationship between the two approaches (p < 0.01, R^2^ = 0.84, Table 2, Fig. 2). An identification threshold of 10% prevalence was observed (Fig. 2), however, 6 samples were identified as 100% aragonite (0% vaterite) by both methods. This suggests that the observed threshold seen was likely due to the detection limits of XRD for small otolith samples, rather than uncertainty in imaged-based estimates of vaterite prevalence.

**Table 2:** Statistical results of a linear model examining the proportions of vaterite in each otolith based on digital image analysis versus X-ray diffraction (XRD).

Factor	DF	SS	MS	F	p	R^2^
XRD	1	5950.4	5950.4	114.6	< 0.001	0.839
Residual	22	1141.3	51.9			
Total	23	7091.7

### Raman spectroscopy

**Figure 3:**
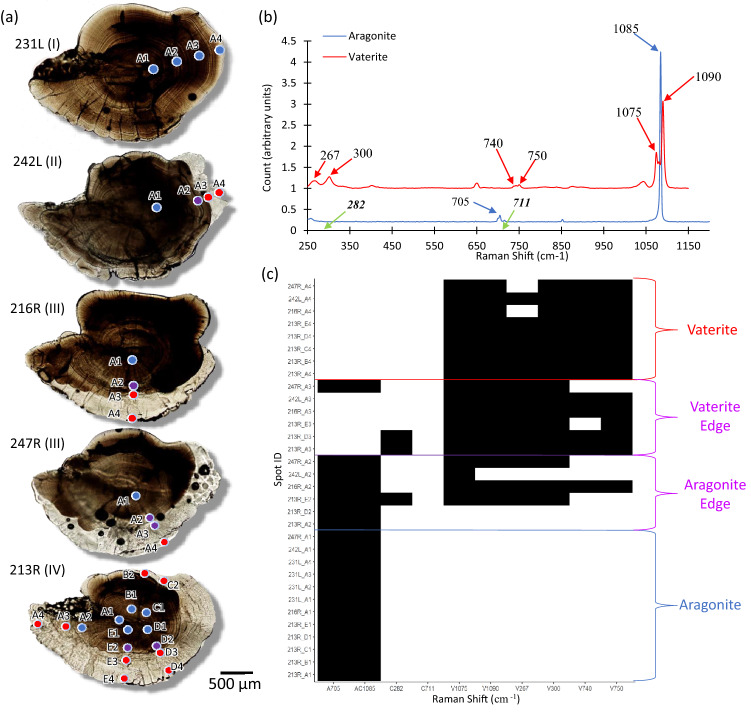
Raman spectroscopy of delta smelt otoliths. (**a**) The five otoliths evaluated for spatial patterns in CaCO_3_ polymorph. Roman numerals indicate the assigned vaterite category. Spots indicate the sampling locations and colors indicate the Raman-detected polymorph (blue-aragonite, red-vaterite, and purple-mixed). (**b**) Examples of Raman spectral shifts with corresponding peaks used for identification. Peaks for calcite (green) were assessed, but no spots reflected significant calcite. Y-axis units are arbitrary. (**c**) Heatmap showing the presence (black) or absence (white) of diagnostic peaks for each polymorph at each spot. X-axis reflects the polymorph (aragonite—A, calcite—C, vaterite—V) and the respective diagnostic peak (cm^−1^).

Raman spectra of aragonitic otoliths (category I) exhibited peaks indicating 100% aragonite (705, 1085 cm^−1^), whereas vateritic otoliths (category II–IV) included peaks indicative of both aragonite and vaterite (267, 300, 740, 750, 1075, and 1090 cm^−1^, Fig. 3b). Peaks at 282 cm^−1^ were observed in 3 otoliths, possibly indicating some calcite; however, no corresponding calcite peak at 711 cm^−1^ was observed, while each spectrum also exhibited multiple peaks indicating the presence of either aragonite or vaterite (Fig. 3c). These spots were therefore classified as either aragonite or vaterite, resulting in limited evidence for the presence of calcite in delta smelt otoliths. Peaks at 155 and 206 cm^−1^ appeared less reliable for discriminating between polymorphs, and were thus excluded from analysis.

### Otolith chemistry

**Figure 4:**
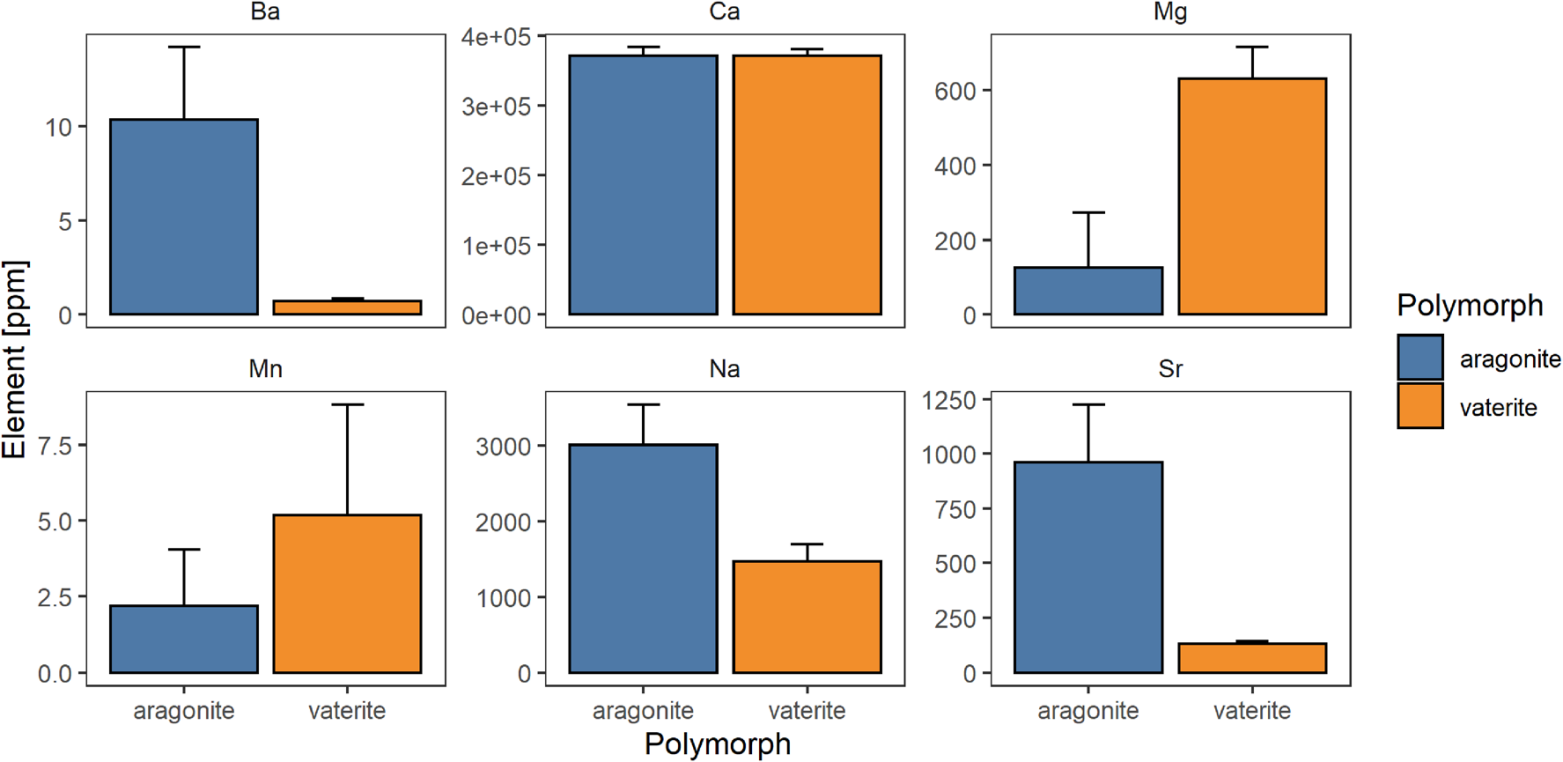
Average elemental concentrations and standard deviation (error bars) between aragonite (blue) and vaterite (orange).

**Figure 5:**
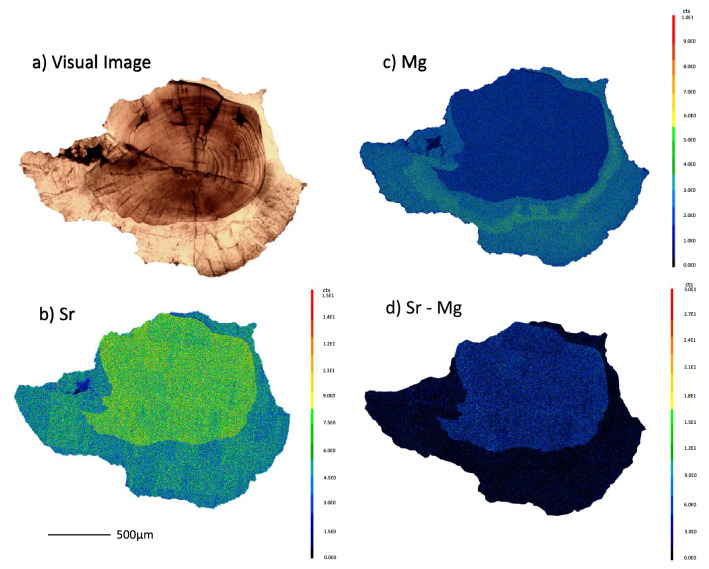
Spatial patterns in otolith polymorphs and chemistry. (**a**) Transmitted light image of a polished delta smelt otolith and contrasting electron microprobe elemental maps of (**b**) Sr, (**c**) Mg, and (**d**) the difference between Sr and Mg. Scale bars are in total counts with ranges varying among plots (**c**,**d**).

We observed significant differences in the elemental compositions of aragonitic and vateritic regions of otoliths. Although Li, K, and Zn were below detection levels, and Ca appeared consistent between crystalline polymorphs, vateritic regions exhibited higher levels of Mg and Mn and lower levels of Na, Ba, and Sr, relative to aragonite. Mg and Mn concentrations were on average 4.6 × and 2.4 × greater in vaterite, while Na, Ba, and Sr were 2x, 14x, and 7.6 × lower in vaterite otoliths, respectively (Fig. 4; Table S1). We did however observe a slight disparity between visual identifications and elemental compositions on otolith aragonite-vaterite edges in Category III and IV otoliths (Fig. S1), likely due to the overlap between polymorphs. Electron probe microanalysis indicated that two-dimensional spatial patterns in the relative abundances of Mg and Sr in otolith sections were consistent with visual patterns in vaterite prevalence and with linear profiles of element-to-calcium ratios based on LA-ICPMS analyses (Fig. 5).

### Vaterite and asymmetry in wild and cultured delta smelt

Vaterite presence and prevalence differed significantly between cultured and wild delta smelt. Greater than 68% of cultured fish contained at least one otolith exhibiting some vaterite (> Category I), compared to just 0.8% of wild fish otoliths (p < 0.001, Tables 1 and 3, Fig. 1). Greater than 18% of cultured fish had at least one otolith exhibiting relatively large amounts (> 15%, category III–IV) of vaterite, whereas no wild fish contained category III–IV otoliths. In total, 32% of cultured fish had no vateritic otoliths, 36% with one vateritic otolith, and 32% with both right and left vateritic otoliths. In contrast, 99% wild fish had no vateritic otoliths, < 1% with one vateritic otolith, and 0% with both right and left vateritic otoliths. Similarly, mean (max) prevalence of vaterite in cultured fish was 7.90%, compared to only 0.05% in wild fish (p < 0.001, Table 3, Fig. S2). Otolith asymmetry varied significantly between wild (1.84%) and cultured (2.40%) fish (p = 0.01, Table 3, Fig. S2). Although hatchery conditions may influence otolith development, the recent 70-days rearing conditions for cultured fish had no detectable effects on the presence (p = 0.268) or prevalence (p = 0.393) of vaterite, nor otolith asymmetry (p = 0.519, Table 3, Fig. S2).

**Table 3:** Results of generalized linear models examining variation in asymmetry and vaterite presence and prevalence in otoliths of wild versus cultured delta smelt (i.e., origin), and among cultured delta smelt as functions of recent (60-days) variation in adult hatchery conditions (*T* temperature, *F* feed), accounting for random tank effects. Significant values are in bold.

Analysis	Model	Null DF	Null residual deviance	Model DF	Model residual deviance	ΔDF	Deviance	p-value
Cultured versus Wild	Asymmetry^†^ ~ Origin	222	87.82	221	85.26	1	2.56	**0.010**
	Vaterite (%) ~ Origin	222	0.17	221	0.45	1	143.46	**< 0.001**
	Vaterite (0,1) ~ Origin	222	293.35	221	160.35	1	132.99	**< 0.001**
Hatchery conditions (cultured)	Asymmetry^†^ ~ T + F + T*F	3	222.78	6	220.51	3	2.27	0.519
	Vaterite (%) ~ T + F + T*F	3	707.06	6	704.07	3	2.99	0.393
	Vaterite (0,1) ~ T + F + T*F	3	132.26	6	128.32	3	3.94	0.268

^†^Square-root transformed.

## Discussion

### Summary of main findings

Here, we quantified the frequency and severity of developmental anomalies in otoliths of delta smelt, a critically endangered estuarine fish that is endemic to the San Francisco Estuary, California, USA. Visual, geochemical, and structural approaches were combined to validate the identification of vaterite in otoliths using digital image analysis. Our study is the first to confirm the presence of vaterite in otoliths of cultured delta smelt, which presented visually as an irregular transparent zone in the otolith and exhibited a distinct signature using LA-ICPMS, XRD, and Raman spectroscopy.

Otoliths of cultured delta smelt were 80 times more likely to contain vaterite (> 1%) and 18 times more likely to contain relatively large (> 15%) amounts of vaterite. Similarly, cultured fish exhibited 1.3 times greater asymmetry than wild fish. Variation in temperature and feed did not appear to affect the presence nor prevalence of vaterite and asymmetry in delta smelt otoliths; however, this may be due in part to the short exposure period of those treatments. In many cases, it was apparent that vaterite formation in otoliths of cultured delta smelt had initiated prior to experimental treatments, suggesting that other culture conditions or longer-term exposures may be responsible for the higher vaterite content in otoliths from the hatchery population. Together, these results indicate that cultured delta smelt exhibit a significantly higher frequency of vestibular abnormalities which can impair hearing, fitness, and survival^21,26,28,58^. Such hatchery effects on otolith development could have important implications for captive culture practices and the supplementation of the wild delta smelt population with cultured individuals.

### Validation

Vaterite in otoliths has previously been quantified using visual^15,59–62^, structural^14^, or geochemical analyses^30,31^. Here, we contrasted results of five different approaches for identifying vaterite in sagittal otoliths of delta smelt. Results based on digital image analysis were highly correlated with those based on XRD, Raman, and geochemical approaches, indicating that visual analysis is a reliable and cost-effective technique for quantifying vaterite in otoliths. For example, 84% of the variation in bulk vaterite content estimated by XRD could be described by visual 2D estimates. Cross referencing spatial patterns in vaterite composition using both Raman spectroscopy and LA-ICPMS with those determined by image analysis further validated the approach. Variation in elemental concentrations as the laser transitioned from aragonite to vaterite regions of the otolith matched expected patterns based on previous studies^16,29,63^. For example, vateritic regions exhibited higher concentrations of magnesium (Mg) and manganese (Mn), and lower concentrations of sodium (Na), barium (Ba) and strontium (Sr) relative to respective aragonitic regions. Results from Raman spectroscopy largely confirmed visual assessments, but also identified mixed aragonite and vaterite signals across aragonite-vaterite edges, suggesting that transitions may be more gradual than they appear visually (Fig. 3).

### Vaterite and asymmetry

The presence of vaterite in cultured (68%) and wild (< 1%) delta smelt otoliths was similar to values observed in cultured and wild populations of other teleost fishes. For example, similar values in cultured populations have been described for Atlantic salmon (66–100%), coho salmon (52–56%), herring (14–60%), rainbow trout (50%), and lake trout (48%)^21^. As for delta smelt, the presence of vaterite in otoliths of wild fish populations is much lower than that of cultured populations, including coho salmon (1–12%), herring (5–6%), rainbow trout (5%), and lake trout (24%)^21^. Overall, vaterite presence is on average 10.4 times higher in cultured fish than wild fish, with up to 91% vaterite prevalence in certain species^16,21^. Otolith asymmetry in wild and cultured delta smelt (1.84–2.40%) was relatively similar to global estimates across numerous taxa (1.25–4.66%)^64^. Hatchery fish have been shown to exhibit up to threefold greater fluctuating asymmetry than wild fish^65^, though this trend is not consistent for all species^66^. Similarly, hatchery delta smelt exhibited 3–4 times higher asymmetry than wild fish, however, even the cultured values remained relatively low.

### Causes of vaterite formation

Vaterite is often naturally occurring in fish otoliths, with its presence varying among taxa and otolith types^14^. For example, sagittal otoliths in sturgeon are largely vateritic, whereas sagittae are aragonitic in most teleost fishes^10^. Within teleosts, sagittae and lapilli are typically comprised of aragonite, whereas asterisci are commonly vateritic^2,9^. The biomineralization of otoliths is tightly regulated through complex patterns in gene expression within the vestibular apparatus and associated variation in protein synthesis and enzymatic activity^9,67–71^. For example, otolith formation in zebrafish is halted when the expression of the *otop1* gene is inhibited^70^. Thus, natural variation in otolith crystalline structures among species, and among otolith types within species, are likely linked to variation in genes and gene expression among taxa and regions of the inner ear of fishes.

Here, we demonstrate that sagittal otoliths of delta smelt can switch from aragonite (normal) to vaterite (abnormal), and that this phenomenon is far more common in cultured versus wild individuals. While the causes of anomalous vaterite formation in otoliths are not fully understood, otolith development appears to be sensitive to environmental conditions and fish bioenergetics. For example, anomalously high levels of vaterite in teleost sagittae have been associated with rapid growth^19^, elevated temperatures^72^, high stocking densities^28^, and handling stress^60^. Similarly, variation in water chemistry may affect otolith biomineralization, with elevated pCO_2_ and reduced pH of ambient water associated with increases in an anomalous (calcite) CaCO_3_ polymorph^73,74^. Changes in [Ca^2+^]/[CO_3_^2−^] within the endolymph is one hypothesized mechanism for stress-induced changes in otolith biomineralization^75,76^. Stress-induced changes in the expression of different otolith macromolecules (e.g., *OMM-64* and *Otolin-1*) may also result in changes in the crystalline structures of otoliths^54,69,77,78^.

### Treatment effects

Although our study was not designed to determine the causes of abnormal otolith development, we nevertheless examined evidence for the effects of temperature, food availability, and their interaction on otolith asymmetry and vaterite formation (Table 3). Here, we detected no significant treatment effects on vaterite or asymmetry in delta smelt otoliths (Table 3). The present study, however, may not fully capture the true nature of environmental and trophic effects on vaterite formation. For example, the short (70-days) duration and limited thermal range of the experimental treatments imposed on cultured fish may have not been sufficient to elicit a measurable effect. Furthermore, the presence of vaterite in some cultured fish prior to their placement in experimental treatments suggests that longer exposures during earlier life stages may result in stronger treatment effects. Targeted experiments that impose longer treatments and wider ranges of culture conditions on younger age-classes may further advance our understanding of the drivers of otolith anomalies in teleosts.

### Consequences of otolith abnormalities

The effects of otolith anomalies on fitness could have important implications for fisheries management and conservation^7,19,28^. For example, the development of vateritic otoliths does not appear to be reversible^21^ and is correlated with several other developmental abnormalities including fewer lateral line neuromasts (i.e., sensory epithelial hair cells), smaller brain weight, and reduced olfactory bulb volume^61^. In adult Norwegian Atlantic salmon, for example, higher frequencies of vaterite corresponded with lower return rates to freshwater rearing habitats^28^, possibly due to reduced sensory function, behavioral alteration, and navigational impairment associated with abnormal otolith development^21,26,27^. As for otolith polymorph anomalies, otolith asymmetry can also impair hearing^25^, cause abnormal swimming behaviors^58^, and limit a fish’s ability to disperse, navigate, and recruit to suitable habitats^79^.

A key concern regarding supplementation of wild populations with cultured fish is whether domesticated populations will exhibit similar fitness and survival as wild populations^80^. The higher prevalence of vaterite observed in cultured delta smelt, for example, may correspond with reduced survival in situ, thus impacting the success of supplementation^28^. For example, altered swimming behavior in delta smelt due to temperature stress leads to higher susceptibility to non-native predators^81^. Furthermore, vestibular and behavioral impairment due to otolith abnormalities may be more common or severe in smaller-bodied fishes such as delta smelt^21,24,27^. The effects of vaterite development on the survival of fish in the wild remains unclear, and effects likely vary ontogenetically^82^. Quantification of the realized effects of vestibular abnormalities in situ remains an important frontier for fisheries management and conservation^7,28,82^.

In addition to physiological effects on fishes, variation in otolith minerology can alter the interpretation of otolith-based metrics^8,32^. This is because polymorph-dependent changes in otolith chemistry can mimic changes associated with environmental variation, such as seasonality or migrations. For example, variation in the minerology of European eel (*Anguilla anguilla*) otoliths resulted in the misidentification of their migratory history^29^. Vaterite in delta smelt sagittae exhibited higher concentrations of Mg and Mn and lower concentrations of Na, Ba, and Sr relative to aragonite, thus proving to be consistent with prior studies, and confirming the importance of otolith minerology for the accurate interpretation of geochemical patterns in otoliths^16,29,63^.

Although vaterite was uncommon in wild delta smelt, the significant quantities of vaterite observed in cultured delta smelt could distort the interpretation of patterns in accretion and geochemistry of hatchery-reared delta smelt. This is particularly concerning given that supplementation of the wild population remains a key conservation strategy. However, most of the vaterite observed in cultured delta smelt was present only on rostral, ventral, and postrostral lobes of the otolith, with the dorsal lobe remaining mostly free of any vaterite. Thus, results from studies that utilize the dorsal lobe of delta smelt otoliths are likely less susceptible to variation in otolith minerology^33,35,44,51^. The quantification of such patterns is key to developing robust methods in otolith research^8,32^.

## Conclusion

Here we quantified the prevalence of otolith crystalline anomalies and asymmetry in wild and cultured populations of delta smelt, a critically endangered estuarine fish that is currently supported by a conservation hatchery. By integrating multiple analytical techniques, we demonstrated that digital image analysis is an effective tool for the rapid quantification of vaterite in fish otoliths. Both vaterite and asymmetry were elevated in otoliths of cultured fish relative to those from the wild population. Although the causes of these anomalies are not fully understood, our results suggests that either (a) hatchery conditions increase otolith abnormalities in cultured fish or (b) that such abnormalities negatively affect fitness and survival, thus leading to strong selection in the wild. Further studies are needed to identify causal mechanisms of vaterite formation in delta smelt and how such abnormalities might affect the hatchery population and the effectiveness of population supplementation efforts.

## Supplementary Information

Supplementary Information.

## Data Availability

All data generated and analyzed during this study are available from the corresponding author upon request.
