# Supplementary material for: Visual, spectral, and microchemical quantification of crystalline anomalies in otoliths of wild and cultured delta smelt
Source: Sci Rep. 2022 Dec 1;12:20751. doi: 10.1038/s41598-022-22813-w (PMC9715569; doi:10.1038/s41598-022-22813-w)
Supplement: Supplementary file 1 — Supplementary Information. [file 41598_2022_22813_MOESM1_ESM.pdf]

SUPPLEMENTARY INFORMATION

Table S1 Concentrations (in ppm) of each element measured in visually identified aragonitic and vateritic regions of each otolith. Values are mean (s.d.).

[X] (ppm)	Aragonite	Vaterite
Na	3012.59 (525.07)	1464.98 (230.26)
Mg	124.97 (147.40)	630.10 (85.34)
Ca	3.71E+05 (1.30E+04)	3.71E+05 (9.60E+03)
Mn	2.19 (1.86)	5.18 (3.63)
Sr	961.97 (260.70)	130.31 (12.96)
Ba	10.37 (3.83)	0.73 (0.15)

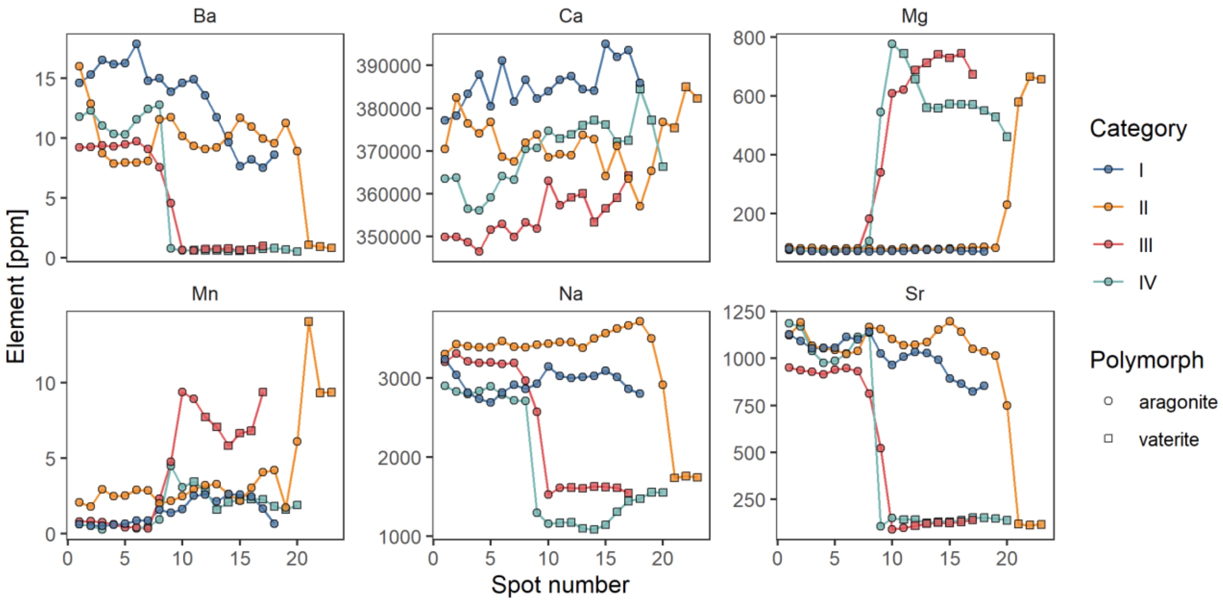

Figure S1. Changes in elemental concentration found between different CaCO3 polymorph aragonite (circle) and vaterite (square) in samples containing varying levels of vaterite replacement (I-IV).

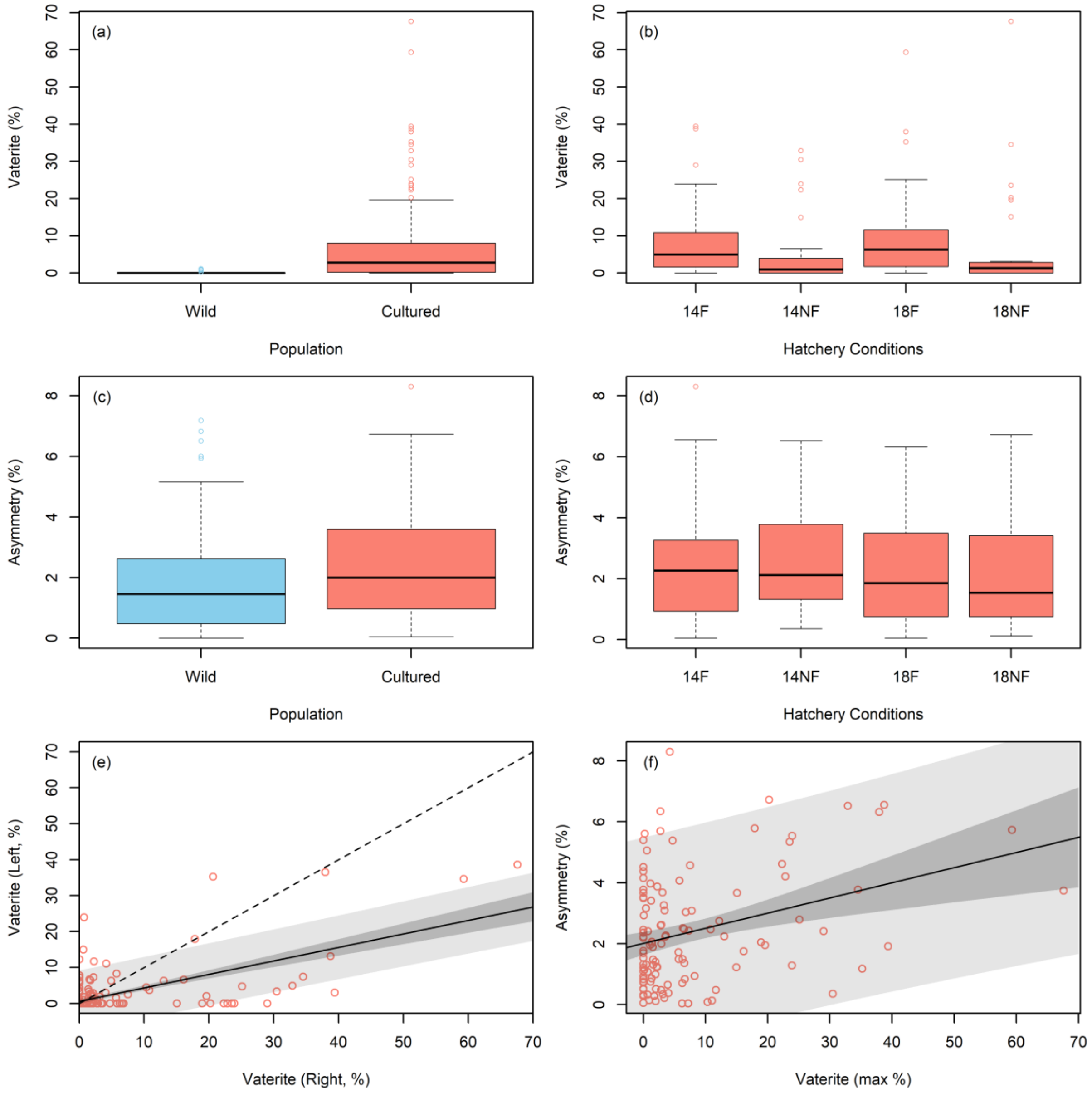

Figure S2. Average vaterite prevalence per fish by (a) origin (b) hatchery condition and the average asymmetry per fish by (c) origin and (d) hatchery condition. The (e) comparison of vaterite prevalence by fish between the left and right otolith and (f) maximum vaterite prevalence and asymmetry.
